# Active compounds: A new direction for rice value addition

**DOI:** 10.1016/j.fochx.2023.100781

**Published:** 2023-07-06

**Authors:** Zhaoqiang Jin, Shaobing Peng, Lixiao Nie

**Affiliations:** a Sanya Nanfan Research Institute of Hainan University, Hainan University, Sanya 572025, China; b MOA Key Laboratory of Crop Ecophysiology and Farming System in the Middle Reaches of the Yangtze River, College of Plant Science and Technology, Huazhong Agricultural University, Wuhan, Hubei 430070, China

**Keywords:** Rice, Active compound, Healthy, Storage, Processing

## Abstract

The development of rice active compounds is conducive to improving the added value of rice. This paper focused on the types and effects of active compounds in rice. Furthermore, it summarized the effect of rice storage and processing technology on rice active compounds. We conclude the following: Rice contains a large number of active compounds that are beneficial to humans. At present, the research on the action mechanism of rice active compounds on the human body is not deep enough, and the ability to deeply process rice is insufficient, greatly limiting the development of the rice active compound industry. To maximize the added value of rice, it is necessary to establish a dedicated preservation and processing technology system based on the physicochemical properties of the required active compounds. Additionally, attention should be paid to the development and application of composite technologies during the development of the rice active compound industry.

## Highlights

• Increasing rice added value contributes to securing world food and economic security.
• Rice active compounds are beneficial for maintaining human health.
• The effects of different processing methods on rice active compounds are reported.
• Rice active compounds have been used successfully in many fields.
• A dedicated processing technology system for rice active compounds is needed.

## 1 Introduction

With the development of the social economy, people's living standards are improving, and they are paying increasing attention to the nutritional and health benefits of food. “Not just eating enough, but more importantly eating well” has become the general consensus. However, along with economic development, people's living environment is deteriorating, and lifestyles have changed significantly, which has had several adverse impacts on human health. The incidence of various modern chronic diseases is increasing, and the proportion of the population in a state of subhealth is growing, which not only puts pressure on healthcare system of society but also brings endless suffering to people (Graham et al., 2001). People are becoming increasingly aware of the functionality and safety of food.

Rice is one of the most important food crops worldwide (He et al., 2021, Jin et al., 2023a, Jin et al., 2023b, Jin et al., 2021) and a major source of income for rural populations (Choudhary et al., 2020). Ensuring the stability of rice production is crucial for maintaining world food and economic security. However, the low economic benefits of planting rice have negatively affected the farmers’ interest to grow rice. Improving rice's added value and hence increasing farmers' income not only enhance farmers' motivation to grow rice, but also assist ensure global food and economic security (Jin et al., 2020).

Rice containing certain specific compounds that play a regulatory and balancing role in human physiological functions in addition to the nutrients necessary for human growth and development in the endosperm, embryo, and rice bran. These compounds boost human physiological defense mechanisms, prevent certain diseases, help recovery, delay aging, and boost physical strength and energy levels (Chen, 2016). Active compounds in rice have a great potential to be exploited for human welfare and health. The total annual production of rice in the world was 950 million tons (Cao et al., 2018), providing sufficient raw materials for the production of rice active compounds. However, the current research and development of active compounds in rice was seriously insufficient, resulting in a serious waste of resources. Therefore, the development of the rice active compound industry has huge advantages. This paper introduced the types and effects of active compounds in rice. Furthermore, it summarized the effects of storage and processing technologies on the content of active compounds in rice. Additionally, we also pointed out the problems existing in the current development of the rice active compound industry and proposed solutions.

## 2 Active compounds in rice and their effects on human health

**Fig. 1:**
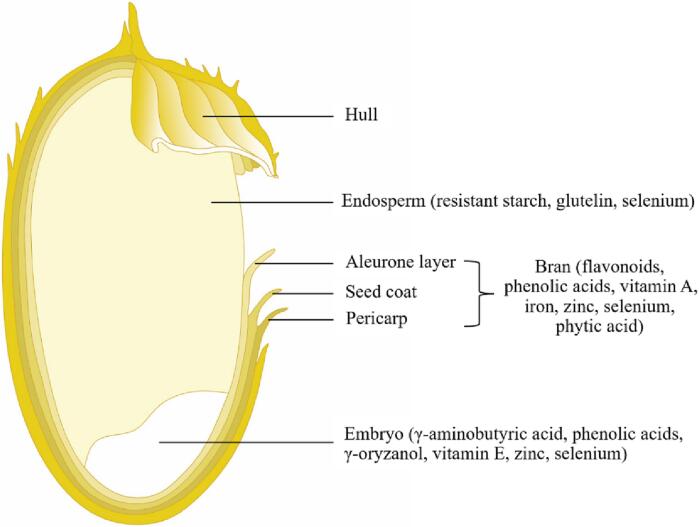
Rice grain structure and the location of its active compounds.

Rice grain consists of the glumes (brown rice) and the hull surrounding the glumes (Zhang et al., 2005) (Fig. 1). Brown rice consists of rice bran, embryo, and endosperm (Zhang et al., 2005). Rice bran surrounds the embryo and endosperm and accounts for about 8–10% of the mass of brown rice, but it contains more than half of the total nutrient content (Yokoyama, 2004). The endosperm is rich in starch and protein and is the main part of the food. The embryo accounts for about 2–3 % of the mass of brown rice and is enriched with a variety of physiologically active compounds (Van Hoed et al., 2006). The common active compounds in rice and their effects on the human body are given in Table 1.

**Table 1:** Rice active compounds and their effects.

Active compounds	Efficiency	References
Resistant starch	Decrease cholesterol; prevention of colon cancer, obesity, diabetes, metabolic disorders and cardiovascular diseases; inhibit the proliferation of tumor cells	Ashwar et al., 2016, Fuentes-Zaragoza et al., 2010, Sharma et al., 2008
Flavonoids	Antibacterial, antiviral, antiallergic, antithrombotic, anti-inflammatory, anticancer and antioxidant; prevention of obesity, type II diabetes, cardiovascular diseases, and atherosclerosis	Bhat et al. (2020); Chen et al., 2016b, Verma and Srivastav, 2020
Phenolic acids	Antiallergic, anticancer, anti-inflammatory, antimicrobial, antithrombotic; prevention of cancer, cardiovascular disease and diabetic complications; decrease cholesterol; protect heart; prevent the accumulation of superoxide and reduce oxidative stress	Walter and Marchesan (2011); Yu et al. (2020)
γ-aminobutyric acid	Anticancer; prevention of neurological diseases, obesity and type II diabetes; decrease blood pressure and blood lipid; enhance kidney and liver function; treatment of brain injury and stroke sequelae	Poojary et al., 2017, Baranzelli et al., 2018
γ-oryzanol	Prevention of obesity and diabetes; inhibit tumor growth; decrease cholesterol; treatment of neurological and menopausal diseases; prevention of cell membrane degradation	Masuzaki et al. (2019)
V_A_	Prevention of dry eye and blindness; maintenance of immunity, spermatogenesis and normal embryo growth	Mason et al., 2005, Wiseman et al., 2017
V_E_	Anticancer, anti-inflammatory, antithrombotic; improve immunity; decrease blood lipid and cholesterol; prevention of cardiovascular and neurodegenerative diseases and diabetes; protect heart; delay the progression of Alzheimer's disease	Sookwong and Mahatheeranont, 2018, Wysota et al., 2017
Fe	Prevention of iron deficiency anemia; maintain immunity; promote intellectual and spiritual development of children	Murray-Kolb & Beard (2009); Shaw et al. (2011)
Zn	Maintenance of nerve and psychological functions and immunity; prevent delivery difficulties for pregnant women, fetal growth retardation and deformities, and anorexia, slow growth and mental retardation in children	Black et al., (2013). (2013)., Hotz and Brown, 2004
Se	Maintain immunity; prevention of cancer, Keshan disease, Kashin-Beck disease and cardiovascular disease; antiaging	Cao et al., 2001, Richie et al., 2006, Wallace et al., 2009
Phytic acid	Reduce starch digestibility and glycemic response; antiaging	Lee et al., 2015, Depar et al., 2013

### 2.1 Resistant starch

Resistant starch (RS) refers to starch that can resist digestion by enzymes in the small intestine and is fermented in the colon (Birt et al., 2013) and is mainly found in the endosperm of rice. Fermentation of RS in the colon produces large amounts of short-chain fatty acids such as acetate, propionate, butyrate, isobutyrate, valerate, and isovalerate (Hung et al., 2016). Short-chain fatty acids have been shown to lower human cholesterol levels and the risk of diabetes and prevent colon cancer, obesity, metabolic disorders, and cardiovascular disease (Ashwar et al., 2016). Butyric acid is also the primary source of energy for colonic epithelial cells and can inhibit the malignant transformation of colonic epithelial cells (Fuentes-Zaragoza et al., 2010). Butyrate can inhibit the proliferation and growth of tumor cells by blocking an important phase of the cell cycle (G1) (Sharma et al., 2008). In addition, as RS cannot be digested, foods with high RS content have lower calories and glycemic index and can be used as a supplement for obese and diabetic patients (Rochfort & Panozzo, 2007).

### 2.2 Flavonoids

Flavonoids are mainly found in the rice bran layer of rice (Hou et al., 2013). Flavonoids have antibacterial; antiviral, antiallergic, antithrombotic (Bhat et al., 2020); anti-inflammatory; anticancer; and antioxidant effects (Verma & Srivastav, 2020). Additionally, it also has preventive effects on obesity; type II diabetes, cardiovascular disease (Chen et al., 2016b); and atherosclerosis. Anthocyanins/proanthocyanidins are the main functional compounds of colored rice (Goufo & Trindade, 2014). Anthocyanins are extremely unstable and rarely exist as free anthocyanins in nature but often exist as glycosides (Zhang et al., 2010).

### 2.3 Phenolic acids

Phenolic acids are mainly found in rice bran and embryos (Verma & Srivastav, 2020). Phenolic acids in rice exist in soluble and insoluble forms, and soluble phenolic acids have free and conjugated forms (Shao et al., 2014). Among the three phenolic acids fractions, insoluble bound phenolic acids are the most abundant, followed by soluble conjugated phenolic acids, and soluble free phenolic acids are the least abundant (Shao et al., 2014). Bound phenolic acids in colored rice accounted for more than 83% of the total phenolic acids (Huang & Lai, 2016). Phenolic acids are natural antioxidants, which reduce oxidative stress and prevent cancer, cardiovascular diseases, and complications of diabetes (Walter & Marchesan, 2011). These also have antiallergy, anticancer, anti-inflammatory, antimicrobial, and antithrombotic effects and prevent superoxide accumulation, reduce cholesterol levels, and protect the heart (Yu et al., 2020).

### 2.4 γ-aminobutyric acid

γ-aminobutyric acid (GABA) is a non-protein amino acid that mainly exists in rice embryos (Liao et al., 2013). GABA is the primary inhibitory neurotransmitter in humans, which helps to maintain the health of the human central nervous system, prevents neurological diseases, relieves depression, and improves sleep quality (Poojary et al., 2017). It also inhibits cancer cell proliferation and stimulates apoptosis of cancer cells (Baranzelli et al. 2018), and improves brain injury and stroke sequelae (Zhang et al., 2005). In addition, GABA has been shown to enhance kidney and liver function, stimulate insulin synthesis in the pancreas, prevent type II diabetes and obesity, and lower blood pressure and lipid levels (Poojary et al., 2017).

### 2.5 γ-oryzanol

γ-oryzanol is mainly found in rice embryos and is one of the main antioxidants in rice. Its antioxidant activity is more than four times that of V_E_ (Laokuldilok et al., 2011). γ-oryzanol inhibits pancreatic β-cell apoptosis, regulates insulin and glucose secretion, and prevents diabetes, but also reduces blood cholesterol levels and prevents the formation of stones (Masuzaki et al., 2019). Additionally, γ-oryzanol also has the effects of preventing obesity, inhibiting tumor growth, preventing cell membrane degradation, and treating nerve imbalance and menopausal diseases (Masuzaki et al., 2019).

### 2.6 Vitamins

Vitamins are a large family, with as many as 13 vitamin compounds known to be required from the human diet (Fitzpatrick et al., 2012). However, V_A_ and V_E_ are the only two vitamins that are widely deficient in people's diet, and have been systematically studied as important active compounds in rice due to their effects on human health.

V_A_ is a group of fat-soluble retinoids, mainly found in rice bran. β-carotene is a precursor compound for the synthesis of V_A_ and can be efficiently converted to retinol in the human body (Tang et al., 2009). V_A_ plays an important role in light transmission, and V_A_ deficiency can cause Xerophthalmia that can lead to complete blindness (Mason et al., 2005). V_A_ deficiency can also damage the immune system of pregnant women and children and even lead to death (Tang et al., 2009). Moreover, V_A_ is also necessary for spermatogenesis and normal embryo growth (Wiseman et al., 2017).

V_E_ mainly found in rice bran and the embryo. The V_E_ content in the embryo is five times that in rice bran (Yu et al., 2007). V_E_ is one of the main antioxidants in the human body and consists mainly of eight congeners of α-, β-, γ-, and δ-tocopherols and α-, β-, γ-, and δ-tocotrienols (Fritsche et al., 2017). The antioxidant capacity of tocotrienols is 40–60 times higher than that of tocopherols, and γ-tocotrienol is the major form of V_E_ in rice (Shammugasamy et al., 2015). Tocotrienols have a better pharmacological potential than tocopherols. It displays anticancer, anti-inflammatory, and antithrombotic properties; has hypolipidemic and cholesterol-lowering effects; and plays a role in preventing diabetes and cardiovascular diseases (Sookwong & Mahatheeranont, 2018). Moreover, V_E_ can also delay the development of Alzheimer's disease in patients, and V_E_ deficiency can aggravate or even induce neurodegenerative diseases (Wysota et al., 2017).

### 2.7 Trace elements

Trace element deficiency is a major health problem in the world today, and trace element malnutrition is commonly known as “hidden hunger.” At present, more than 2 billion people worldwide suffer from different forms and degrees of micronutrient deficiencies (FAO, 2019). Fe, Zn, and Se are the three trace elements that have the greatest impact and are most often deficient in the human body. Moreover, these trace elements that are currently the focus of functional rice biofortification.

Fe in rice is mainly found in the aleurone layer, and its deficiency can cause anemia and lead to death in severe cases (Shaw & Friedman, 2011). Fe deficiency can also cause complications such as impaired intellectual and mental development in children, weakened immunity, and reduced work capacity in adults (Murray-Kolb & Beard, 2009).

Zn is mainly located in the embryo and aleurone layer of rice, with very low concentrations in the endosperm. Approximately 10% of proteins in biological systems require Zn to maintain their structural and functional integrity (Andreini et al., 2006). It has been shown that Zn deficiency causes a decrease in neuropsychological function and immunity in humans and also leads to delivery difficulties in pregnant women, fetal growth retardation, and malformations. It also plays a role in anorexia, slow growth, mental retardation in children, and even death in severe cases (Black et al., 2013). In addition, Zn deficiency can impair taste, smell, and memory and affects spermatogenesis in adults (Hotz & Brown, 2004).

More than half of the Se in rice exists in the endosperm. The endosperm and rice bran mainly contain the organic and inorganic forms of Se, respectively (Liang et al., 2018). Organic Se is biologically more accessible to humans than inorganic Se (Longchamp et al., 2015). Selenium helps maintain human immunity, reduces the risk of cancer, and prevents cardiovascular disease (Wallace et al., 2009). Selenium deficiency can cause Keshan and Kashin-Beck diseases (Cao et al., 2001). Clinical studies have also shown that Se supplementation can increase intracellular concentrations of the antioxidant glutathione (GSH) and the activity of its synthesis-limiting enzyme γ-glutathione cysteine ligase. It can reduce tissue and blood concentrations of oxidants and slow human aging (Richie et al., 2006). However, it is important to note that excessive Se intake can adversely affect human health (Longchamp et al., 2015).

### 2.8 Phytic acid

Phytic acid (PA) mainly exists in rice bran. It is highly reactive and can bind divalent mineral cations (e.g., Fe, Zn, and Ca) to form mixed salt complexes, resulting in reduced bioavailability of these nutrients (Depar et al., 2013). Increasing the Fe content in rice endosperm without reducing the level of PA did not significantly increase the absorption of Fe by humans (Lee et al., 2015). Similarly, the molar ratio of phytate to Zn is a valuable parameter to evaluate the bioavailability of Zn, and the molar ratios of phytate to Zn are 4–15 and 61–74 for polished and brown rice, respectively (Ma, 2007). Jou et al. (2012) indicated that Zn uptake decreased with an increase in molar ratio of PA to Zn. When the molar ratio of PA to Zn exceeds 15, less than 15% of the Zn in food can only be absorbed and utilized by the body. However, PA is also beneficial to humans, as the combination of PA and Fe can reduce lipid peroxidation and free radical production and improve the antioxidant properties of rice (Lee et al., 2015). PA can reduce starch digestibility in vitro and the glycemic response in vivo by binding proteins closely related to starch digestion or digestive enzymes that are proteins themselves (Depar et al., 2013).

## 3 Effect of storage on the active compounds of rice

Many active compounds in rice are chemically unstable, and rice hulls can greatly protect active compounds in rice against environmental influences, making rice grains more storage-friendly than rice (Norkaew et al., 2017). High temperature, light, and oxygen will accelerate the decomposition of active compounds in rice. Storing rice at low temperature and light intensity or in nylon / LLDPE bags under vacuum or inert gases can significantly extend the storage period of rice (Ge et al., 2021; Norkaew et al., 2017). Rice embryos contain large amounts of free fatty acids, which are highly susceptible to oxidative rancidity, producing off-flavor compounds, and reducing the content of active compounds such as phenolic compounds and flavonoids in rice (Zhou et al., 2014). The oxidative activity of rice can be reduced by reducing the content of free fatty acids, increasing the level of antioxidants, and reducing lipase activity. This can prolong the storage time and improve the quality of stored rice (Zacheo et al., 2000). A pre-cooking treatment inactivates the lipase in rice, slows down lipid oxidation, and allows the penetration of some minerals and water-soluble vitamins from the bran into the endosperm. However, the pre-cooking process can also destroy the antioxidant compounds in rice, thereby reducing the antioxidant properties of rice (Jaisut et al., 2009).

After harvest, rice should be protected from sun exposure and high-temperature drying. It should preferably be air-dried in a cool, dry, and ventilated place and stored with the husk intact in a low temperature, low humidity, low oxygen, and low light intensity environment. For rice with active compounds that are not biologically active, pre-cooking, drying, and insecticidal sterilization treatment can be carried out prior to storage.

## 4 Effect of processing on the active compounds of rice

The active compounds in rice are mainly found in the rice bran and embryo, with low levels of active compounds in the endosperm. In milling rough rice into polished rice, the rice bran and embryo, and most of the nutrients, are removed. However, due to the poor palatability of brown rice, consumption is much lower than that of refined rice (Ohtsubo et al., 2005). Therefore, establishing a dedicated processing technology system that can improve the function and edible quality of rice is one of the important links to promoting the development of the rice active compound industry. Nevertheless, there are limited studies on this aspect, the following is a detailed review of the dry and wet processing technologies used for brown rice.

### 4.1 Dry processing technology

The widely used dry processing technologies for brown rice include high-pressure, pulsed electric fields, plasma, and irradiation treatment. Dry processing is easier than wet processing, and the technical requirements and equipment prices are lower. Even though it is convenient to promote the application, its effect on improving brown rice’s nutritional value and palatability are limited.

#### 4.1.1 High-pressure processing

High**-**pressure (HP) destroys the structure of the pericarp and aleurone layer of brown rice, thereby improving its palatability and cooking characteristics. The degree of destruction depends on the intensity and duration of the applied pressure. HP can also disrupt cell integrity and protein structure, enabling the release of allergenic proteins from brown rice (Boluda-Aguilar et al., 2013). However, direct HP treatment of germinated brown rice will accelerate lipid hydrolysis and oxidation and cause browning compounds during the storage of brown rice (Hashimoto, 2020), which affects the commercial quality of rice. In contrast, HP treatment of brown rice before germination can increase GABA content, delay lipid hydrolysis and oxidation, keep brown rice color stable, and extend its storage period (Xia & Li, 2018). Additionally, HP treatment inactivates microorganisms and enzymes in brown rice. A moderate pressure maintains enzyme activity and leads to partial cell breakdown. This allows enzymes to more readily reach target substrates in the germinated brown rice matrix and promotes biotransformation reactions leading to the production of large amounts of bioactive compounds such as polyphenols, GABA, flavonoids, and V_E_ (Xia et al., 2019).

#### 4.1.2 Pulsed electric fields processing

The main effect of a pulsed electric fields (PEF) treatment is to inactivate the microorganisms and enzymes in brown rice and has little effect on its sensory and nutritional properties. The inhibition effect is mainly related to frequency, pulse width, retention time, and voltage (Wu et al., 2019). PEF treatment can also improve the permeability of bran by altering its microstructure, thus increasing the rate of material transfer (Zhao et al., 2014). In addition, the extraction of active compounds in brown rice can be greatly enhanced by treating brown rice with PEF and corresponding extraction solvents (Quagliariello et al., 2016).

#### 4.1.3 Plasma processing

The process of gas ionization produces a large number of chemically active compounds that can induce modification of food chemical composition and enzyme and microbial inactivation (Misra et al., 2016). Plasma treatment can break down the bran of brown rice, resulting in faster water absorption, reduced cooking time, reduced hardness, and improved palatability (Potluri et al., 2018). The results of Chen et al. (2015) showed that plasma treatment could inhibit the activities of enzymes such as α-amylase, lipase, and lipoxygenase; delaying starch denaturation, lipid hydrolysis and oxidation in brown rice, and resulting in the extended shelf life of brown rice. Mild plasma treatment can be used to increase the content of active compounds in germinated rice products. Chen et al. (2016a) found that low-pressure plasma shock waves significantly increased the germination rate, α-amylase activity, GABA and total phenolic compounds content, and antioxidant capacity of brown rice.

#### 4.1.4 Irradiation processing

Food irradiation is mainly for health purposes by inactivating microorganisms and insects. Low-intensity γ-irradiation does not cause significant changes in starch, protein, and lipid contents, but high-intensity γ-irradiation affects biomolecules’ structure and physical and chemical properties (Sung, 2005). Studies have found that γ-irradiation can partially break the covalent bonds between phenols and glucose, leading to an increase in phenolic content and antioxidant capacity of brown rice, and corresponding with the irradiation dose (Shao et al., 2013). γ-radiation can also increase the content of RS in brown rice by producing β- (1–3) and β- (1–4) bonds in starch through transglycosylation (Rombo et al., 2004). Ultraviolet (UV) irradiation inhibits the growth of aerobic bacteria, yeasts, and fungus and improves the stability of brown rice, but also leads to the degradation of GABA (Ferreira et al., 2021). Too low irradiation intensity will affect the sterilizing effect, and excessive irradiation will lead to browning and flavor changes, reducing its commercial value. Moderate irradiation treatment will help delay starch aging during storage and extend the shelf life of brown rice (Sung, 2005).

### 4.2 Wet processing technology

The widely used wet processing technologies for brown rice include germination, fermentation, pre-cooking, ultrasonic, and enzyme treatment. The effect of wet processing on the nutritional value and palatability of brown rice is higher than that of dry processing. However, the technical requirements of the process, the risk of spoilage, and the drying cost after processing are higher, which greatly limits its usage and application.

#### 4.2.1 Germination

Brown rice is germination is induced by first soaking the rice. Compared to ungerminated brown rice, germinated brown rice has a softer texture, higher sweetness, better taste, and better cooking characteristics (Ng et al., 2013) and is, therefore, popular among consumers. Additionally, a large number of hydrolytic enzymes were activated during germination, resulting in a significant increase in the content of bioactive compounds such as dietary fiber, free fatty acids, GABA, folic acid, vitamins, γ-oryzanol, flavonoids, and phenolic acids; and micronutrients such as Ca, Mg, Fe, and Zn (Kaur et al., 2017), while the content of anti-nutritional compounds was significantly reduced, thereby greatly improving the nutritional value of brown rice (Table 2). However, nutritional loss occurs when the rice soaked for a long time before germination. In addition, under favorable temperature and nutritional conditions, microorganisms will proliferate rapidly, which will lead to brown rice spoilage and produce unpleasant odors that will seriously affect the commercial quality of rice. Heat treatment is known to inhibit microbial growth (Bari et al., 2009), however, this process severely affects the germination of seeds. Using electrolyzed water as a detergent and soaking agent can effectively kill bacteria and promote brown rice germination and GABA accumulation (Liu et al., 2013), while a 0.5% lactic acid solution can effectively overcome the odor generated during germination process (Thitinunsomboon et al., 2013). However, the current selection of inexpensive, safe, and efficient soaking agents is limited.

**Table 2:** Effect of germination on the content of active compounds in rice.

Sample	Treatment	γ-aminobutyric acid content (mg 100 g^−1^)	γ-oryzanol content (mg 100 g^−1^)	Phenolic acids content (mg GAE 100 g^−1^)	References
Polished rice		1.70	6.10		Ohtsubo et al. (2005)
Brown rice		6.04	48.20		
Germinated brown rice	Soak in distilled water for 24 h and germinate for 24 h	11.02			
	Soak for in distilled water 24 h and germinate for 48 h	27.73			
	Soak for in distilled water 24 h and germinate for 72 h	69.21	50.40		
	Soak in distilled water for 24 h and germinate for 96 h	149.03			
Brown rice		12.00	8.06		Roohinejad et al. (2010)
Germinated brown rice	Soak and germinate in distilled water for 24 h	20.00	9.02		
	Soak and germinate in distilled water for 48 h	44.00	13.60		
Germinated brown rice	Soak in distilled water for 6 h and germinate for 20 h	22.15			Hussain et al. (2020)
	Soak in distilled water for 6 h and germinate for 40 h	38.78			
	Soak in distilled water for 6 h and germinate for 40 h	36.85			
	Soak in distilled water for 6 h and germinate for 20 h	20.95			
Brown rice		1.07	11.17	132.53	Cáceres et al. (2017)
Germinated brown rice	Soak in distilled water for 24 h	12.75	14.08	118.14	
	Soak in distilled water for 24 h and germinate for 48 h	36.41	14.09	190.29	
	Soak in distilled water for 24 h and germinate for 96 h	49.85	18.18	359.22	
Germinated brown rice	Soak in electrolyzed oxidizing water for 84 h	16.25			Lu et al. (2010)
	Soak in 1% NaClO solution for 84 h	7.91			
	Soak in alkaline electrolyzed water for 84 h	10.52			
	Soak in distilled water for 84 h	9.09			
Brown rice		11.99			Wei et al. (2013)
Germinated brown rice	Soak in distilled water for 10 h and germinate for 24 h	32.08			
	Soak in 0.5 g/L FeSO_4_ solution for 10 h and germinate for 24 h	30.86			
	Soak in 1 g/L FeSO_4_ solution for 10 h and germinate for 24 h	21.65			
	Soak in 2 g/L FeSO_4_ solution for 10 h and germinate for 24 h	16.38			
Rough rice		8.26		57.65	
Germinated rough rice	Soak in distilled water for 24 h	10.70		66.61	Cáceres et al. (2014)
	Soak in distilled water for 24 h and germinate for 48 h	80.70		114.04	
	Soak in distilled water for 24 h and germinate for 96 h	107.48		252.16	
Germinated brown rice	Soak in distilled water for 3 h and germinate for 8 h		6.60		Ng et al. (2013)
	Soak in distilled water for 3 h and germinate for 12 h		6.69		
	Soak in distilled water for 3 h and germinate for 16 h		17.70		
	Soak in distilled water for 3 h and germinate for 20 h		18.10		

Notes: GAE represent gallic acid equivalents.

#### 4.2.2 Fermentation

The metabolism and enzymatic transformation by microorganisms during fermentation can improve the bioavailability of micronutrients in rice and reduce the content of anti-nutritional factors. Fermentation increases the content of fiber, minerals, phenols, V_E_, and antioxidant properties in brown rice (Kradangar & Songsermpong, 2015) and decreases the content of PA and the molar ratio of PA to Zn (Liang et al., 2008). Se can also be biotransformed into the organic form during brown rice fermentation (Lanfear et al., 1993).

#### 4.2.3 Pre-cooking

The pre-cooking process consists of three steps: soaking, cooking and, drying. Pre-cooking improves the textural properties of brown rice and inhibits denaturing enzymes, thereby extending the shelf life of brown rice and expanding the availability of brown rice products. It also increases the content of RS and reduces the content of PA (Liyanaarachchi et al., 2021) in brown rice. However, high-temperature heating can also cause the degradation of active compounds in rice and increase the occurrence of rice browning, which reduces the commercial value of rice (Mir et al., 2016). The pre-cooking process is time-consuming, laborious, and energy-consuming, limiting the application thereof.

#### 4.2.4 Ultrasound processing

In recent years, ultrasound has been widely used in food processing (Ding et al., 2018). The current application in brown rice processing is mainly low-intensity ultrasound, which does not significantly damage the structure and affect the cooking characteristics of brown rice but increases the germination rate and GABA accumulation (Xia et al., 2020). The power of high-intensity ultrasoundcan significantly destroy the structure of rice and promote the hydrolysis and oxidation of lipids during storage. After high-intensity ultrasonic treatment, the cooking time of brown rice is shortened, and the texture is softened (Ding et al., 2018). However, ultrasonic treatment leads to the loss of active compounds in rice (Peanparkdee et al., 2018). Therefore, attention should be paid to the recovery of active compounds in the liquid medium when processing rice with ultrasound.

#### 4.2.5 Enzyme processing

Treating brown rice with enzymes can degrade the bran layer and improve texture and cooking characteristics. For instance, the treatment of brown rice with xylanase and cellulase not only increased the germination rate and GABA level (Jia et al., 2016) but also made it easier for water to diffuse into grains, thus reducing cooking time. The phenolic compounds and mineral content remained largely unchanged after enzyme treatment (Geng et al., 2020). Pullulanase or isoamylase can debranch amylopectin in brown rice, increase the amylose content, and improve the content of RS in rice by retrogradation (Trinh et al., 2013). However, if the reaction time is too long or the enzyme dosage is too high, the short-chain (DP < 10) produced by debranching will inhibit the crystallization of amylose, thereby reducing the content of RS (Trinh et al., 2013). Brown rice processed with pectinase has better taste and higher nutrient retention rate (Pih & Kim, 2013).

From the above, it is clear that both dry and wet rice processing technologies have advantages and disadvantages. Therefore, the application of composite technology in rice processing should be paid attention to improve the appearance and storage quality of rice while increasing the content of active compounds in rice in the future.

## 5 Development of rice active compound related products

At present, rice active compound products have been applied to many fields such as food, healthcare and cosmetics. Value-addition of rice and its by-products will help increase the income of rice farmers, improve their motivation to grow rice, and ensure global food security. It can also provide functional products for most consumers, achieving a win–win situation for both rice producers and consumers.

### 5.1 Application in the field of food

#### 5.1.1 Rice products

Rice is used as a staple food, and many rice products have been developed. The U.S. Department of Agriculture has developed Ricemic, a modified rice starch food with delayed digestion, reducing the glycemic load and offering benefits to diabetic patients (Mukamuhirwa et al., 2019). Distiller's grains made from brown rice are conducive to treating obesity (Kim et al., 2011). Wine, vinegar, and beverages made from colored rice have strong antioxidant capacities (Takeshita et al., 2016), and colored compounds extracted from colored rice have been used as food coloring, replacing artificial colors (Zhang et al., 2010). The active compounds in rice have a wide range of applications, but the current development of rice active compounds in the field of food is far from sufficient, and relevant research needs to be strengthened.

#### 5.1.2 Rice bran products

Rice bran is the most important by-product of brown rice processing. Although it only accounts for 6–8% of the mass of brown rice, it contains 60–70% of the total physiologically active compounds of brown rice (Park et al., 2017). It contains a range of bioactive compounds such as γ-oryzanol, phytosterols, vitamins, flavonoids, polyphenols, squalene, arabinoxylan, polysaccharide alcohol, PA, and ferulic acid, and micronutrients such as Fe, Zn, Se, Cu, and Mg (Park et al., 2017). Additionally, rice bran from colored rice contains high amounts of anthocyanins. However, a lack of knowledge of active compounds has led to its usage mainly as animal feed for a long time, resulting in wasted resources. Improving the comprehensive utilization value of rice bran is an effective way to improve the added value of rice, which has attracted widespread attention.

Rice bran can be used as a food additive. The addition of 20% rice bran to flour used for bread making improves the antioxidant activity and storage stability of bread (Irakli et al., 2015) without affecting its quality. The addition of rice bran to cookies improves the texture of cookies and extends the shelf life (Bhanger et al., 2008). The antioxidant activity and polyphenols, flavonoids, and anthocyanin content were higher in noodles supplemented with rice bran (Kong et al., 2012). Loypimai et al. (2017) found that adding 2.5% rice bran fiber to Frankfurt sausage can improve its texture and consistency while reducing its bond force, delaying the oxidation of lipids, and prolonging its storage time. Solid beverages made from rice bran nutrients and rice bran nutritional fiber are rich in protein, unsaturated fatty acids, vitamins, dietary fiber, and minerals. They have a unique flavor and low price, offering strong market potential (Pham et al., 2017).

Whole fat rice bran contains 18–22% oil. Rice bran oil contains more than 80% unsaturated fatty acids (Bhat et al., 2020) and is a healthy edible oil. Long-term consumption of rice bran oil reduces the absorption of cholesterol in the body, enhances the removal of cholesterol, and reduces the risk of kidney stones (Wallace et al., 2016). It also prevents cardiovascular diseases, scavenges free radicals that damage the human immune system, and strengthens human immunity (Baschieri & Cortelli, 2019).

### 5.2 Application in the field of healthcare

The application of rice in healthcare is mainly reflected in the application of rice bran. Rice bran is rich in a variety of bioactive compounds that can improve human body functions. Flavonoids have various biological effects such as antibacterial, anti-inflammatory, and antiallergic and have been used in a clinical setting as anti-inflammatory and antiallergic agents (Bhat et al., 2020). γ-oryzanol can reduce atherosclerosis and inhibit platelet aggregation (Reena et al., 2010), and the pharmaceutical industry has developed drugs against cardiovascular disease with γ-oryzanol as the main ingredient. Arabinoxylan is a potent biological response modifier that activates natural killer cells, T cells, and monocytes and also inhibits the progress of HIV (Ghoneum & Agrawal, 2011), which has been widely used in the treatment of related diseases. Rice bran polysaccharide has anti-tumor, anti-radiation, and anti-oxidation effects; lowers blood fat and sugar levels; enhances immunity and scavenges free radicals (Pang et al., 2018) and a variety of drugs have been studied around these effects. In addition to inhibiting lipid peroxide production in the human body, PA enhances human immunity, has anti-cancer properties, and has been widely used in the health care industry (Norhaizan et al., 2011). Rice bran of colored rice is also rich in anthocyanins and has potent free radical scavenging activity (Buonocore et al., 2012), which can slow down human aging and has attracted the attention of many nutraceutical companies. In addition, oral hydrolyzed rice bran can prevent common cold syndrome in the elderly (Maeda et al., 2004); antibiotics such as oxytetracycline, cephalosporin, and bacteriocin (Sawa et al., 2010) can also be extracted from solid fermented rice bran.

### 5.3 Application in the field of cosmetics

Rice bran contains many compounds that are beneficial to human skin. γ-oryzanol inhibits UV-induced lipid peroxidation and can be used as a sunscreen, to eliminate wrinkles and lessen hyperpigmentation. Ferulic acid and its esters can stimulate hair growth and reduce the risk of atopic dermatitis in humans (Burlando & Cornara, 2014). Squalene is a natural moisturizer that softens the skin, effectively delays wrinkle formation, protects the skin from sun damage, and maintains a healthy complexion (Bhat et al., 2020). Rice bran wax can promote ceramide synthesis and activate glutathione reductase, affecting skin revitalizing and anti-aging (Sabale et al., 2007). Proanthocyanidins in colored rice bran inhibit melanin production and the cross-linking of skin connective tissue by collagenase and elastase, thus improving skin immunity and maintaining skin elasticity (Buonocore et al., 2012).

## 6 Problems and solutions in the development of the rice active compound industry

Comprehensive research on rice active compounds is required to maintain people's health and is needed for market and rice industry development. However, there are still many obstacles in the current development of the rice active compound industry.

Firstly, there is a lack of active compound-specific rice varieties that can be planted on a large scale. The innovation of germplasm resources is the driving force behind the development of the rice active compound industry. The limitation of breeding technology, genetic resources, gene patents, and variety rights has seriously affected the breeding of active compound-specific rice varieties, resulting in the lack of active compound-specific rice varieties that can be promoted and planted on a large scale, which seriously limits the development of the rice active compound industry.

Secondly, there is a lack of supporting cultivation control technology system. The cultivation control technology is an essential basis for the smooth and healthy development of the rice active compound industry. The research on rice active compounds is still in its infancy, and related scientific research is limited, and the existing research is also more disorganized and has not yet formed a system.

Thirdly, the research on the regulation mechanism of active compounds synthesis in rice and the mechanisms involved in maintaining human health is limited. Clarifying these aspects is necessary for the efficient and rational utilization of rice active compounds.

Fourthly, the capacity to process rice is insufficient. Different active compounds are located in different parts of rice and have different effects on the human body. Therefore, research on different processing technology systems needed for different active compounds in rice is required.

Given the above problems, we believe that future efforts to develop the rice active compound industry should focus on the following areas:

The first aspect is strengthening the research of active compound-specific rice breeding technology and developing close-linked markers related to active component synthesis genes, thereby enriching the genetic resources of active compound-specific rice breeding. At the same time, a platform for exchange and cooperation of active compound-specific rice breeding technology and functional compound synthesis genes should be established to break the barriers of breeding technology and resources, maximize the use of effective technology and genetic resources, and ultimately achieve the goal of breeding by design.

The second aspect is strengthening research on active compound-specific rice cultivation and regulation technology, establishing supporting cultivation technology systems for different active compound-specific rice, using technology to guide production, and promoting the transformation of scientific research results.

The third aspect is strengthening multidisciplinary synergistic research; conducting comprehensive and in-depth identification and analysis of active compounds in rice from various aspects such as physiology, nutrition, molecular biology, medicine, and food science; clarifying the regulatory mechanism of rice functional compound synthesis and the mechanisms involved in maintaining human health; and laying a solid foundation for the efficient utilization of rice active compounds.

The fourth aspect is developing and promoting the deep processing technology of rice and its by-products, extending the rice active compound industrial chain, and improving the added value of rice while increasing policy and economic support for the production and research of rice active compounds and promoting the integrated development of rice active compounds production and research.

## 7 Conclusion

Rice contains many active compounds that are beneficial to maintaining human health and have high economic and social value with broad market prospects. However, the current rice processing technology is not perfect. Rice processing technologies that improve both the palatability and active compound content of rice tend to reduce rice's appearance and storage quality, as well as having high processing costs and technical constraints that make commercialization challenging. The processing technology with lower processing costs and technical requirements has a single effect on improving the palatability and functional compound content of rice, and the improvement effect is not obvious. Furthermore, the current application of rice active compounds is still relatively narrow, owing to the current high cost of rice active compound production, the low popularity of rice active compound goods, and people's failure to fully realize the value of rice active compounds. Therefore, research on rice preservation and processing technologies should be strengthened, and a preservation and processing technologies system for rice dedicated to active compounds that can improve the edible and nutritional quality of rice while reducing processing costs and technical requirements should be established in the future. Simultaneously, the variety and potential value of rice active compounds should be further explored to increase the added value of rice and promote the healthy and sustainable development of the rice active compound industry.

## CRediT authorship contribution statement

**Zhaoqiang Jin:** Conceptualization, Data curation, Formal analysis, Investigation, Methodology, Software, Validation, Visualization, Writing – original draft, Writing – review & editing. **Shaobing Peng:** Conceptualization, Supervision, Writing – review & editing. **Lixiao Nie:** Conceptualization, Formal analysis, Funding acquisition, Project administration, Supervision, Validation, Writing – review & editing.

## Declaration of Competing Interest

The authors declare that they have no known competing financial interests or personal relationships that could have appeared to influence the work reported in this paper.

## Data Availability

Data will be made available on request.
